# Monochorionic-specific association between first-trimester serum ferritin and gestational diabetes in twin pregnancies: a retrospective cohort study

**DOI:** 10.3389/fendo.2025.1616668

**Published:** 2025-10-24

**Authors:** Yanyan Ni, Yan Bi, Xiaona Xu, Yucheng Hu, Jue Ma, Yanlin Wang

**Affiliations:** School of Medicine, Shanghai Jiao Tong University, International Peace Maternity and Child Health Hospital, Shanghai, China

**Keywords:** serum ferritin (SF), gestational diabetes mellitus (GDM), twin pregnancy, chorionicity, monochorionic diamniotic (MCDA)

## Abstract

**Background:**

Previous studies have demonstrated that elevated serum ferritin (SF) levels in early pregnancy are significantly associated with the risk of developing gestational diabetes mellitus (GDM). However, these findings have primarily focused on singleton pregnancies, and evidence in twin pregnancies remains underexplored. This study aimed to explore the association between early-pregnancy SF levels and the risk of GDM in twin pregnancies, with a particular focus on different chorionicity types.

**Methods:**

We conducted a retrospective cohort study involving 882 twin pregnancies delivered at our hospital between January 2019 and December 2021. The cohort included 700 dichorionic diamniotic (DCDA) and 182 monochorionic diamniotic (MCDA) pregnancies. Cases with gestational age at delivery less than 28 weeks, pre-existing diabetes, unknown GDM status, or mid-trimester fetal reduction in monochorionic-triamniotic (MCTA) pregnancies were excluded. GDM was diagnosed using a 75 g oral glucose tolerance test (OGTT) based on the IADPSG criteria. Serum ferritin (SF) levels were measured during the first prenatal visit in the first trimester. Logistic regression, linear correlation analyses and Receiver Operating Characteristic (ROC) curve were performed to assess associations between SF and GDM.

**Results:**

In MCDA pregnancies, women with GDM had significantly higher mean SF levels compared to those without GDM (101.68 ± 59.72 vs. 79.87 ± 53.11 μg/L, p<0.05). However, no significant difference was observed in DCDA pregnancies. In MCDA cases, SF levels >71.4 μg/L were independently associated with an increased risk of GDM (adjusted OR = 2.775, 95% CI: 1.191–6.466; p=0.018), with a significant trend across SF levels (p for trend = 0.012). Additionally, SF was positively correlated with fasting blood glucose in early pregnancy (r=0.17, p=0.025) and 1-hour OGTT glucose at 24–28 weeks (r=0.15, p=0.041) among MCDA pregnancies.

**Conclusions:**

Elevated SF levels in early pregnancy are independently associated with a higher risk of GDM in MCDA twin pregnancies and may serve as a potential early biomarker for GDM prediction. In contrast, no significant association was found in DCDA pregnancies, indicating that the predictive value of SF may differ by chorionicity. Further studies are warranted to confirm these findings and investigate the underlying mechanisms.

## Introduction

Gestational diabetes mellitus (GDM) is a common pregnancy complication, defined as glucose intolerance with onset or first recognition during pregnancy (1). It is known to significantly elevate the risk of maternal and fetal complications, particularly in twin pregnancies, which are inherently associated with increased metabolic demand and placental complexity (2).

Accumulating evidence from experimental and clinical studies suggests that GDM is essentially a state of chronic insulin resistance, largely mediated by proinflammatory cytokines that impair insulin signaling and reduce insulin secretion from pancreatic β-cells (3, 4). In this inflammatory milieu, iron metabolism plays a pivotal role. Iron, a redox-active transition metal, can catalyze the formation of reactive oxygen species (ROS) when present in excess (5). These ROS promote oxidative stress, which in turn exacerbates insulin resistance and impairs β-cell function, ultimately contributing to the pathogenesis of GDM (6, 7).

High body iron stores have been consistently associated with increased diabetes risk in multiple epidemiological studies (8–12). Serum ferritin (SF), the primary intracellular iron-storage protein, is also an acute-phase reactant. Its circulating levels rise not only in response to iron overload but also under inflammatory conditions (13, 14). Elevated SF levels may further propagate the inflammatory response, leading to pancreatic β-cell dysfunction, heightened insulin resistance, and β-cell exhaustion, and may even contribute to hepatic insulin resistance and glucose dysregulation (15, 16). These pathophysiological changes eventually impair glucose uptake by skeletal muscle and promote hepatic gluconeogenesis, facilitating the development of diabetes (17).

As a result, numerous studies have investigated SF as a potential biomarker for GDM, and a consistent positive association has been observed between elevated SF levels in early pregnancy and subsequent GDM development in singleton pregnancies (12, 13, 18–25). Based on these findings, early-pregnancy SF levels are now recognized as a potential predictive marker for GDM in singleton gestations (26).

Twin pregnancies are associated with a higher incidence of GDM (3-9% morbidity statistically) (27–32), early prediction can help us identify and reduce its morbidity. But there is a noticeable lack of biochemical markers predicting the risk in this specific population. Studies have found that a certain proportion of GDM may likely result from the same pathogenesis as the singleton pregnancy: greater transient increase in insulin resistance (33, 34), therefore we could definitively establish the early predictive utility of SF in twin gestations. However, different types of twins have distinct hemodynamic changes, inflammatory responses and placental number due to the different chorionicity, which might lead to different mechanisms for GDM. We should evaluate the utility of SF particularly with respect to chorionicity-related differences and various risk factors of GDM.

In our research, we conducted a retrospective cohort study to evaluate the association between early-pregnancy SF levels and the risk of GDM diagnosed according to the criteria of the International Association of Diabetes and Pregnancy Study Groups (IADPSG) in twin pregnancies with different chorionicity. By exploring this relationship, we aim to facilitate earlier identification of high-risk individuals, thereby enabling timely interventions—such as dietary counseling and lifestyle modifications—to reduce GDM-related maternal and perinatal morbidity in the growing population of twin pregnancies.

## Materials and methods

### Study population and sample collections

This retrospective cohort study encompassed all twin pregnancies delivered at our institution in Eastern China from January 1, 2019 to December 31, 2021. A total of 882 eligible cases were identified. The exclusion criteria were: singleton pregnancies; deliveries before 28 weeks of gestation; absence of first-trimester ultrasound data to determine chorionicity or gestational age; twin pregnancies that became monochorionic diamniotic (MCDA) after mid-trimester fetal reduction in monochorionic triamniotic (MCTA) pregnancies; and pre-existing diabetes mellitus.

Upon enrollment, written informed consent was obtained from all participants, the institutional review board approved the study (approval reference number: GKLW-A-2024-023-01), and maternal medical histories were documented. Blood samples were collected during the first prenatal visit in early pregnancy(<12 pregnant weeks, empty stomach, ECLIA, Roche Cobas analyzer, regular calibration using the standards provided by manufacturer) to measure SF levels. Screening for GDM was performed at 24–28 weeks of gestation using a 75 g oral glucose tolerance test (OGTT), and diagnoses were based on the IADPSG criteria: fasting plasma glucose ≥ 5.1 mmol/L, 1-hour glucose ≥ 10.0 mmol/L, or 2-hour glucose ≥ 8.5 mmol/L.

### Data collection

Because of the unique physiologic characteristics of different chorionicity, we divided the included pregnancies into dichorionic diamniotic (DCDA,700 cases) and monochorionic diamniotic (MCDA,182 cases) twins. Chorionicity was initially assessed via prenatal ultrasonography and subsequently confirmed by intraoperative and pathological findings after delivery. Clinical and laboratory data were extracted from the hospital’s electronic medical record system, including maternal demographic characteristics, obstetric and medical histories, and laboratory indices. Gestational age was confirmed based on first-trimester ultrasound.

### Statistical analysis

Continuous variables were summarized as means ± standard deviations (SDs), and categorical variables were reported as frequencies and percentages. Comparisons between the GDM and non-GDM groups were performed using independent samples t-tests for continuous variables and chi-square tests for categorical variables.

Linear regression analyses were conducted to evaluate the associations between serum ferritin levels (as the dependent variable) and potential influencing factors, including maternal age, pre-pregnancy body mass index (P-BMI), geographical residence, educational level, mode of conception, family history of type 2 diabetes, hemoglobin level, HbA1c, fasting plasma glucose in early pregnancy, and OGTT results. These analyses were performed using the R programming language.

To determine the predictive value of SF for GDM, the optimal serum ferritin threshold was identified using the Youden index derived from receiver operating characteristic (ROC) curve analysis. Based on this cutoff, logistic regression models were applied to assess the association between elevated SF and the risk of GDM. Crude and adjusted odds ratios (ORs and aORs), along with their 95% confidence intervals (CIs), were calculated. The significance of trends across SF levels was also evaluated. All statistical analyses were conducted using SPSS software, version 29.0 (IBM Corp., Armonk, NY), and a two-sided p value < 0.05 was considered statistically significant.

Missing data were handled by complete-case analysis at the variable level. When a specific measurement was unavailable for a patient, that patient was excluded only from analyses involving that variable, without excluding the entire patient record. As the overall proportion of missing data was small (<3%), no imputation was performed.

## Results

### Baseline characteristics and early pregnancy SF levels in MCDA and DCDA twin pregnancies

A total of 182 MCDA and 700 DCDA twin pregnancies were included in the analysis. **Tables 1**, **2** show the baseline maternal characteristics stratified by GDM status in MCDA and DCDA groups. **Tables 3**, **4** summarize the early pregnancy laboratory results stratified by GDM status in MCDA and DCDA groups, respectively.

**Table 1:** Demographic differences of women with MCDA pregnancies.

Characteristics	Non-GDM group (n=142)	GDM group (n=40)	p-value
Maternal age (year)	31.57 ± 4.52	32.45 ± 4.33	0.270
Maternal age≥35 years	38 (26.8%)	12 (30%)	0.840
Pre-pregnancy BMI (kg/m2)	21.33 ± 2.72	22.87 ± 3.41	**0.010**
Multiparity	42 (29.6%)	6 (15%)	0.100
Assisted Reproductive Technology (ART) pregnancy	28 (19.7%)	15 (37.5%)	**0.030**
chronic hypertension	1 (0.7%)	3 (7.5%)	0.050
History of GDM	1 (0.7%)	0 (0)	1.000
History of polycystic ovary syndrome(PCOS)	0(0%)	0(0%)	
Family history of type II diabetes	8 (5.6%)	7 (17.5%)	**0.040**
Geography			0.270
Shanghai	55 (38.7%)	20 (50%)	
Foreign/expatriate	87 (61.3%)	20 (50%)	
Educational level			0.720
Bachelor’s degree or above	104 (73.8%)	27 (69.2%)	
specialist degree or below	37 (26.2%)	12 (30.8%)	
Smoking	1 (0.7%)	0 (0%)	1.000
Twin-to-twin transfusion syndrome (TTTS)	8(5.6%)	0(0%)	0.272
Twin anemia–polycythemia sequence (TAPS)	3(2.1%)	0(0%)	1.000
Selective intrauterine growth restriction(sIUGR)	12(8.5%)	2(5%)	0.698

*Bachelor’s degree or above: further study at university after graduating from high school.

*specialist degree or below: further study at college after graduating from high school or below.

Bold values means p-value is < 0.05 with a statistically significant difference.

**Table 2:** Demographic differences of women with DCDA pregnancies.

Characteristics	Non-GDM group (n=548)	GDM group (n=152)	p-value
Maternal age (year)	32.28 ± 3.7	33.3 ± 3.54	**0.002**
Maternal age≥35 years	140 (25.5%)	61 (40.1%)	**<0.001**
Pre-pregnancy BMI (kg/m2)	21.37 ± 2.89	22.22 ± 2.94	**0.002**
Multiparity	57 (10.4%)	19 (12,5%)	0.560
Assisted Reproductive Technology (ART) pregnancy	417 (76.1%)	109 (71.7%)	0.320
chronic hypertension	6 (1.1%)	3 (2%)	0.660
History of GDM	1 (0.2%)	3 (2%)	0.050
History of polycystic ovary syndrome(PCOS)	17 (3.1%)	9 (5.9%)	0.170
Family history of type II diabetes	32 (5.8%)	16 (10.5%)	0.070
Geography			**0.040**
Shanghai	248 (45.3%)	84 (55.3%)	
Foreign/expatriate	300 (54.7%)	68 (44.7%)	
Educational level			
Bachelor’s degree or above	361 (67.1%)	101 (67.8%)	0.950
specialist degree or below	177 (32.9%)	48 (32.2%)	0.950
Smoking	4 (0.7%)	1 (0.7%)	1.000

Bold values means p-value is < 0.05 with a statistically significant difference.

**Table 3:** Blood sampling tests of women with MCDA pregnancies.

Characteristics	Non-GDM group (n=142)	GDM group (n=40)	p-value
Early pregnancy			
Ferritin	79.87 ± 53.11	101.68 ± 59.72	**0.040**
Fasting blood glucose	4.56 ± 0.45	4.66 ± 0.44	**0.240**
Glycated hemoglobin	5.24 ± 0.28	5.42 ± 0.31	0.002
hemoglobin	124.32 ± 10.71	126.62 ± 12.07	0.280
Folic.Acid	34.37 ± 9.33	37.08 ± 11.79	0.190
Vitamin.B12	351.88 ± 127.34	373.97 ± 114.14	0.300
Middle pregnancy (OGTT)			
Fasting blood glucose	4.12 ± 0.43	4.63 ± 0.59	<0.001
1h after	7.6 ± 1.24	10.21 ± 1.27	<0.001
2h after	6.29 ± 1.06	8.93 ± 1.57	<0.001
Glycated hemoglobin	12.71 ± 1.41	12.67 ± 1.15	0.860

*early pregnancy: <12 gestational weeks.

*middle pregnancy(OGTT): OGTT test performed at 24–28 weeks of gestation.

Bold values means p-value is < 0.05 with a statistically significant difference.

**Table 4:** Blood sampling tests of women with DCDA pregnancies.

Characteristics	Non-GDM group (n=548)	GDM group (n=152)	p-value
Early pregnancy			
Ferritin	92.34 ± 70.14	87.79 ± 72.01	0.215
Fasting blood glucose	4.54 ± 0.4	4.66 ± 0.43	0.002
glycated hemoglobin	5.23 ± 0.29	5.33 ± 0.39	0.003
hemoglobin	125.81 ± 9.62	127.51 ± 9.09	0.050
Folic.Acid	34.04 ± 8.38	35.16 ± 9.1	0.180
Vitamin.B12	363.56 ± 133.95	341.27 ± 118.4	0.050
Middle pregnancy (OGTT)			
Fasting blood glucose	4.17 ± 0.37	4.52 ± 0.51	**<0.001**
1h after	7.65 ± 1.19	10.15 ± 1.16	**<0.001**
2h after	6.45 ± 1.04	8.89 ± 1.46	**<0.001**
Glycated hemoglobin	12.73 ± 1.52	13.03 ± 1.49	**0.030**

*early pregnancy: <12 gestational weeks.

*middle pregnancy(OGTT): OGTT test performed at 24–28 weeks of gestation.

Bold values means p-value is < 0.05 with a statistically significant difference.

In MCDA pregnancies, women who developed GDM had significantly higher pre-pregnancy BMI (p = 0.01), a higher proportion of ART-conceived pregnancies (p = 0.03), and a greater frequency of family history of type II diabetes (p = 0.04) compared with non-GDM women. Notably, the mean serum ferritin (SF) level in early pregnancy was significantly higher in the GDM group than in the non-GDM group (101.68 ± 59.72 vs. 79.87 ± 53.11 μg/L, p = 0.04). Early pregnancy HbA1c was also elevated in the GDM group (p = 0.002).

In contrast, in DCDA pregnancies, although GDM was associated with older maternal age (p = 0.002), higher pre-pregnancy BMI (p = 0.002), and increased HbA1c levels (p = 0.003), no significant difference in SF levels was observed between GDM and non-GDM groups (87.79 ± 72.01 vs. 92.34 ± 70.14 μg/L, p = 0.49).

### Association between early pregnancy SF and GDM risk in MCDA pregnancies

To assess the predictive value of SF for GDM, we conducted logistic regression analysis in MCDA pregnancies using the SF threshold of 71.4 μg/L, identified via ROC curve and Youden index.

As shown in **Table 5**, after adjustment for potential confounders (maternal age, parity, history of GDM, family history of diabetes, pre-pregnancy BMI, ART pregnancy, chronic hypertension, smoking, early Hb and HbA1c), women with SF > 71.4 μg/L had a significantly increased risk of developing GDM compared to those with SF ≤ 71.4 μg/L (adjusted OR = 2.775; 95% CI: 1.191–6.466; p = 0.018). A dose-response trend was also observed across SF categories (p for trend = 0.012), supporting a potential threshold effect.

**Table 5:** Association of early pregnancy SF level with GDM risk in MCDA pregnancies.

Variables in the Equation		OR		
	Sig.	Exp(B)	95% C.I.for EXP(B) (Lower)	95% C.I.for EXP(B) (Upper)
Step 1a(maternal age ≥35)	0.572	1.242	0.586	2.633
a. Variable(s) entered on step 1: maternal age≥35				
Variables in the Equation		OR		
	Sig.	Exp(B)	95% C.I.for EXP(B)	95% C.I.for EXP(B)
Step 1a(BMI divided into four groups)			Lower	Upper
Group 1	1.000	1	0.268	3.737
Group 2	0.028	4.978	1.189	20.845
Group 3	0.161	4	0.575	27.819
a. Variable(s) entered on step 1: BMI divided into four groups (BMI<18.5, 18.5≤BMI<24, 24≤BMI<28, BMI≥28).				
Variables in the Equation		OR		
	Sig.	Exp(B)	95% C.I.for EXP(B) (Lower)	95% C.I.for EXP(B) (Upper)
Step 1a	0.037	11.526	1.166	113.952
a. Variable(s) entered on step 1: chronic hypertension(without marked for 0, with marked for 1)				
Variables in the Equation		OR		
	Sig.	Exp(B)	95% C.I.for EXP(B) (Lower)	95% C.I.for EXP(B) (Upper)
Step 1a	1.000	1	0	0
a. Variable(s) entered on step 1: history of GDM(without marked for 0, with marked for 1)				
Variables in the Equation		OR		
	Sig.	Exp(B)	95% C.I.for EXP(B)	95% C.I.for EXP(B)
Step 1a			Lower	Upper
group1	0.067	0.416	0.163	1.063
group2	0.999	0	0	0
a. Variable(s) entered on step 1: parity(unipara marked for 1, delivery once marked for 2, delivery marked twice for 3).				
Variables in the Equation		OR		
	Sig.	Exp(B)	95% C.I.for EXP(B) (Lower)	95% C.I.for EXP(B) (Upper)
Step 1a	0.133	1.706	0.85	3.426
a. Variable(s) entered on step 1: geography (Shanghai marked for 0, foreign/expatriate marked for 1).				
Variables in the Equation		OR		
	Sig.	Exp(B)	95% C.I.for EXP(B) (Lower)	95% C.I.for EXP(B) (Upper)
Step 1a	1.000	1	453661135.8	0
a. Variable(s) entered on step 1: smoking (without marked for 0, with marked for 1).				
Variables in the Equation		OR		
	Sig.	Exp(B)	95% C.I.for EXP(B) (Lower)	95% C.I.for EXP(B) (Upper)
Step 1a	0.636	1.337	0.402	4.441
a. Variable(s) entered on step 1: hemoglobin and glycated hemoglobin level in early trimester (anemia marked for 1, without marked for 0).				
Variables in the Equation		OR		
	Sig.	Exp(B)	95% C.I.for EXP(B) (Lower)	95% C.I.for EXP(B) (Upper)
Step 1a	0.007	2.841	1.325	6.092
a. Variable(s) entered on step 1: SF: 71.4.				
Variables in the Equation		OR		
	Sig.	Exp(B)	95% C.I.for EXP(B) (Lower)	95% C.I.for EXP(B) (Upper)
Step 1a	<.001	9.286	3.401	25.354
a. Variable(s) entered on step 1: glycated hemoglobin level in early trimester:5.65				
Variables in the Equation		OR		
	Sig.	Exp(B)	95% C.I.for EXP(B) (Lower)	95% C.I.for EXP(B) (Upper)
Step 1a	0.009	2.72	1.284	5.761
a. Variable(s) entered on step 1: conception method (natural conception marked for 0, assisted reproductive technology pregnancy marked for 1).				
Variables in the Equation		aOR		
	Sig.	Exp(B)	95% C.I.for EXP(B)	95% C.I.for EXP(B)
Step 1a			Lower	Upper
Group 1	0.945	1.052	0.25	4.421
Group 2	0.081	4.108	0.841	20.067
Group 3	0.512	2.138	0.221	20.687
conception method	0.068	2.271	0.941	5.482
glycated hemoglobin level in early trimester:5.65	0.001	6.324	2.073	19.292
SF:71.4	0.018	2.775	1.191	6.466
a. Variable(s) entered on step 1: BMI divided into four groups, conception method, glycated hemoglobin level in early trimester:5.65, SF:71.4).				
Variables in the Equation		p for trend		
	Sig.	Exp(B)	95% C.I.for EXP(B)	95% C.I.for EXP(B)
Step 1a			Lower	Upper
conception method	0.008	3.08	1.338	7.091
BMI group median	0.026	1.205	1.022	1.42
Glycated hemoglobin group median	0.026	5.933	1.241	28.37
ferritin group median	0.012	1.01	1.002	1.018
a. Variable(s) entered on step 1: conception method, BMI group median, glycated hemoglobin group median, ferritin group median.				

**Figure 1:**
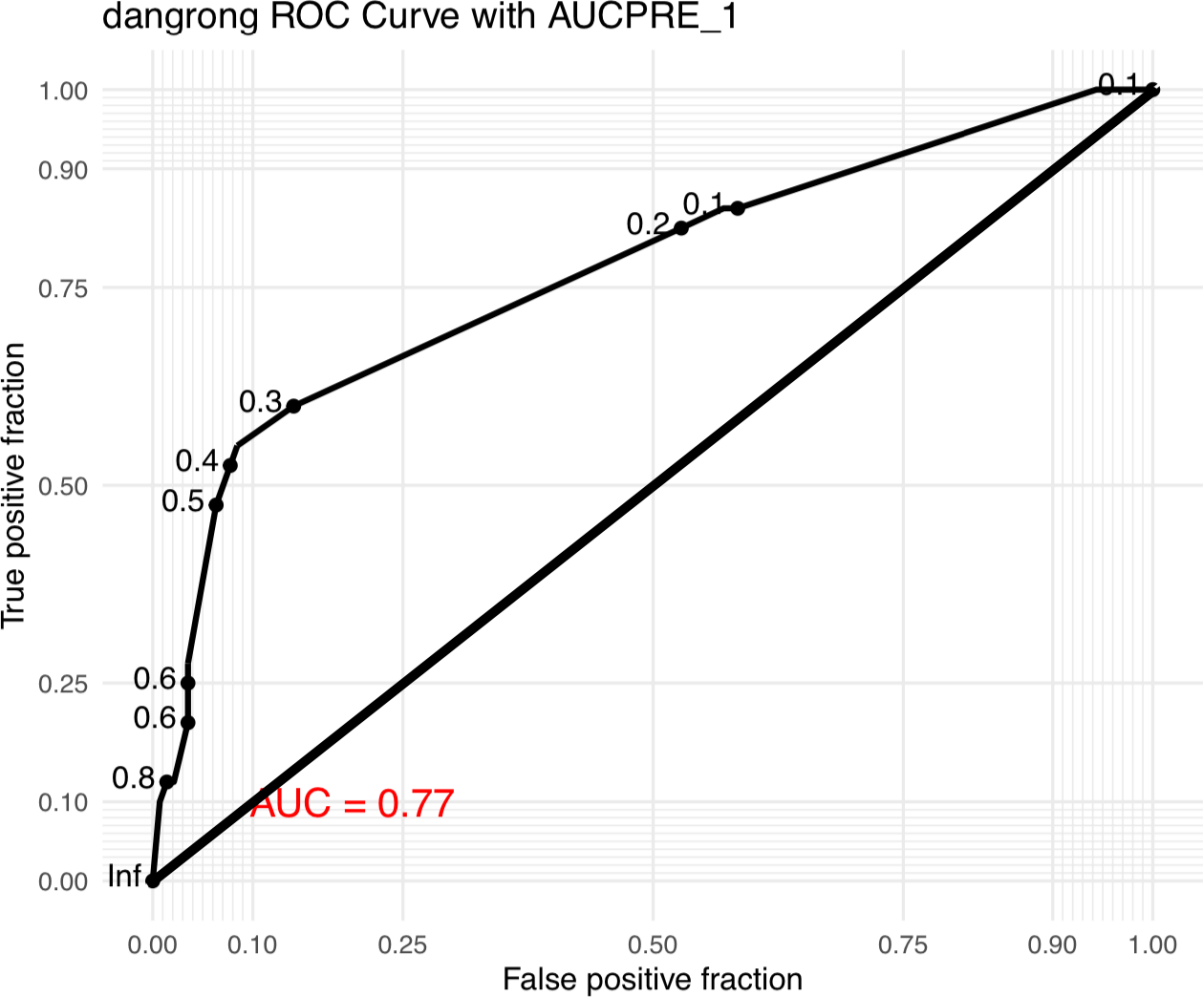
The ROC curve of GDM prediction model in MCDA pregnancies.

The ROC curve of the prediction model of GDM in MCDA pregnancy was shown in **Figure 1** (area under curve:0.77).The value of SF>71.4μg/L was found to be 72.5% sensitive and 50.7% specific. At the cutoff value, calculated positive predictive value and negative predictive values are 29.3% and 86.7% respectively.

### Correlations between SF and glycemic parameters in MCDA pregnancies

**Figure 2:**
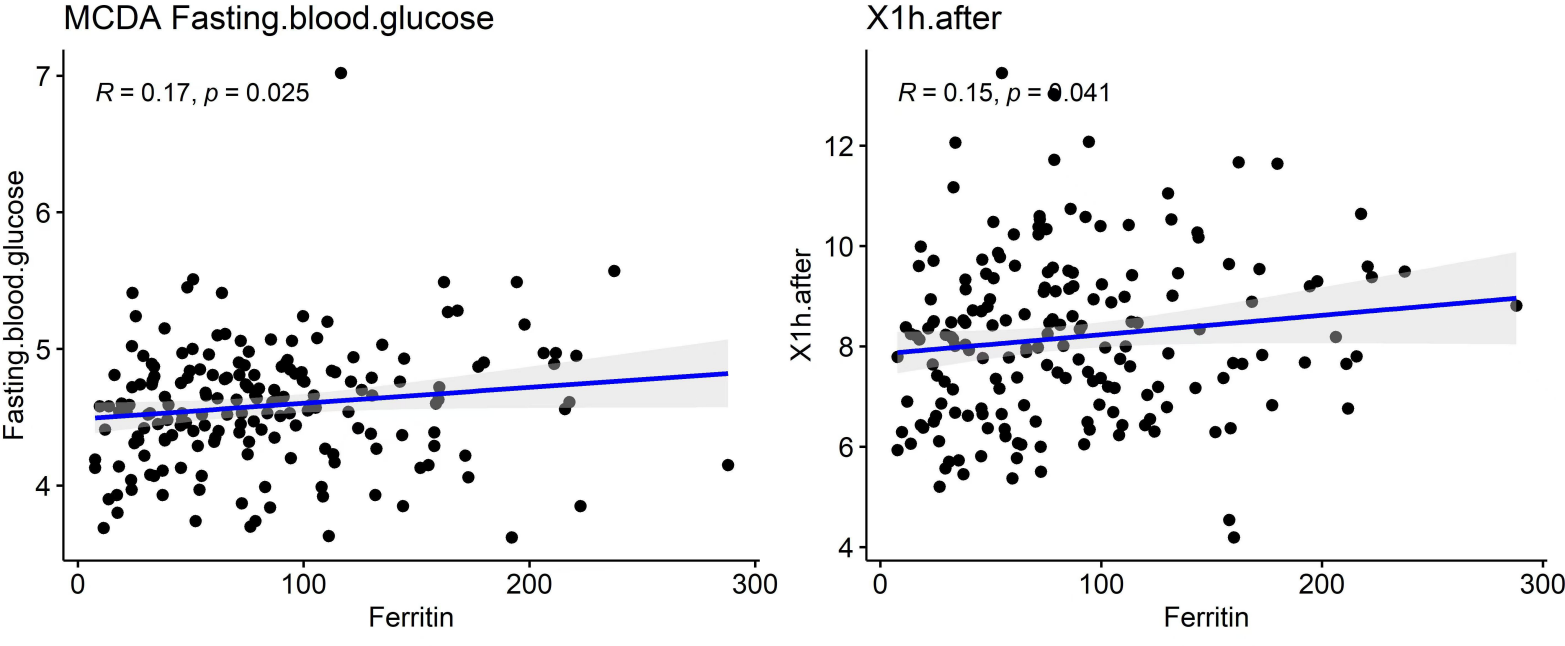
Correlations of SF between fasting blood glucose in early pregnancy and 1-h OGTT level.

To further explore the metabolic significance of serum ferritin, correlation analysis was performed between early pregnancy SF and glucose-related indices. As shown in **Table 6** and **Figure 2**, SF levels were positively correlated with fasting blood glucose in early pregnancy (r = 0.17, p = 0.025) and 1-hour OGTT glucose at 24–28 weeks (r = 0.15, p = 0.041).

**Table 6:** Correlations between SF and the characteristics of the MCDA pregnancy women in a simple correlation model.

Characteristics	Serum ferritin	
	r	p-value
Maternal age (years)	-0.12	0.110
Pre-pregnancy BMI (kg/m2)	0.0038	0.960
Geography(%)	-0.14	0.062
Educational level(%)	0.091	0.230
Conception method(%)	-0.11	0.140
Family history of type II diabetes(%)	-0.009	0.900
Hemoglobin in early pregnancy(g/l)	0.044	0.550
Glycated hemoglobin in early pregnancy(%)	0.084	0.260
Fasting blood glucose in early pregnancy (mmol/L)	0.17	**0.025**
Fasting blood glucose of OGTT(mmol/L)	0.14	0.052
1h after(mmol/L)	0.15	**0.041**
2h after(mmol/L)	0.073	0.330
Glycated hemoglobin (%)	-0.08	0.280

Bold values means p-value is < 0.05 with a statistically significant difference.

No significant correlations were observed with maternal age, pre-pregnancy BMI, or early pregnancy hemoglobin levels. These results indicate that elevated SF may be associated with early alterations in glucose metabolism in MCDA pregnancies.

Taken together, these results demonstrate that elevated serum ferritin levels in early pregnancy are significantly associated with increased risk of GDM in MCDA twin pregnancies, but not in DCDA pregnancies. This association remains significant after adjustment for key clinical risk factors and correlates with both early and mid-gestation glycemic indices.

## Discussion

In this retrospective observational study, we found that elevated SF in early pregnancy was significantly associated with an increased risk of GDM in MCDA pregnancies but not in DCDA pregnancies. After adjusting for key confounding factors, including maternal age, parity, history of GDM, family history of diabetes, pre-pregnancy BMI, assisted reproductive technology pregnancy, chronic hypertension, smoking, hemoglobin and glycated hemoglobin level in early trimester, high SF remained an independent predictor of GDM in the MCDA group. Moreover, early-pregnancy SF levels were positively correlated with fasting glucose and 1-hour OGTT glucose levels, indicating a potential link between iron metabolism and glucose dysregulation in MCDA pregnancies. So we can conclude that with the measurement of SF we can predict the risk of development of GDM even before its development.

Several potential mechanisms may explain the association between elevated SF and GDM risk, particularly in MCDA twins. Unlike DCDA pregnancies, where two fetuses develop independently from separate ova and have distinct placentas, MCDA twins originate from a single fertilized ovum and share a common placenta, with a similar maternal inflammatory response like the single pregnancy, the greater increase in insulin resistance was observed (due to the greater placental mass) (33–37). Our findings consistent with previous studies conducted in singleton pregnancies further confirm the point. For instance, Cheng et al. (22) and Liu et al. (38) demonstrated that elevated SF in early pregnancy was significantly associated with impaired glucose tolerance and subsequent GDM especially linearly correlated with 1-hour OGTT. Notably, we observed a significant linear relationship between SF and 1-hour OGTT levels, which aligns with evidence suggesting that the 1-hour glucose value is more closely linked to insulin resistance and β-cell dysfunction than fasting or 2-hour values (39, 40). Conversely DCDA twins have two separate placentas, the interaction between two placental factors might result in the hemodynamic changes and inflammatory responses completely different.

In MCDA twins, unique complications such as twin-to-twin transfusion syndrome (TTTS), selective intrauterine growth restriction (sIUGR), and twin anemia–polycythemia sequence (TAPS) are more frequent. These conditions may result in dynamic fluctuations in fetal and maternal hemoglobin levels, stimulating hepatic ferritin synthesis as a compensatory response. This increase in ferritin may reflect a state of subclinical inflammation or metabolic stress, both of which are known contributors to impaired insulin sensitivity and increased GDM risk (13–16).

Taken together, our results support the hypothesis that SF is not only a passive marker of iron status but also an active participant in the pathogenesis of GDM, particularly in MCDA pregnancies where placental structure and oxidative stress levels may amplify its impact. Early identification of high SF levels may allow clinicians to stratify GDM risk in twin pregnancies more precisely and implement timely interventions to reduce adverse outcomes.

Moreover, we identified a positive correlation between SF and fasting plasma glucose in the first trimester. Physiologically, insulin sensitivity is typically enhanced in early pregnancy to support maternal–fetal nutrient delivery, resulting in lower fasting glucose levels. However, elevated SF may contribute to early-onset insulin resistance, thereby blunting this adaptive mechanism and raising fasting glucose levels. This suggests that we should pay more attention to fasting glucose with increasing SF level in the early trimester, early screening and intervention when necessary.

Despite the strengths of our study, including a large sample size and stratified analysis by chorionicity, several limitations should be acknowledged. First, due to its retrospective nature, we could not obtain accurate data on dietary iron intake or iron supplementation, which may influence SF levels and confound associations. Second, SF concentrations were measured only in the first trimester, and dynamic changes in iron status during pregnancy were not captured. Third, we acknowledge that the MCDA GDM sample size is limited (n=40), leading to wide CIs. The proportion of MCDA in twin pregnancies is relatively low, especially in cases of GDM in MCDA, we only collected 40 cases during the two-year period. Given the limited sample size, the findings should be regarded as preliminary and exploratory, need to be further validated with more cases. Besides, we relied on a single biomarker (SF) rather than a panel of iron metabolism or inflammatory indicators, which limits the mechanistic interpretation of our findings. Future prospective studies incorporating broader iron indices and inflammatory markers are warranted to further elucidate these associations.

## Conclusion

In conclusion, our study demonstrates that elevated serum ferritin in early pregnancy is independently associated with increased risk of GDM in MCDA twin pregnancies. SF may serve as a cost-effective and accessible early biomarker to predict GDM in this high-risk population, potentially guiding individualized screening and preventive strategies. In contrast, no such association was observed in DCDA pregnancies, highlighting the importance of considering chorionicity in the metabolic evaluation of twin gestations.

## Data Availability

The original contributions presented in the study are included in the article/supplementary material. Further inquiries can be directed to the corresponding authors.
